# Mechanisms and Strategies to Overcome Drug Resistance in Colorectal Cancer

**DOI:** 10.3390/ijms26051988

**Published:** 2025-02-25

**Authors:** Jennifer Haynes, Prasath Manogaran

**Affiliations:** Department of Clinical and Translational Sciences, Joan C. Edwards School of Medicine, Marshall University, 1600 Medical Center Drive, Huntington, WV 25701, USA; prasathmanogaran@gmail.com

**Keywords:** colorectal cancer, drug resistance, chemotherapy, targeted therapy, immunotherapy, resistance mechanisms, therapeutic strategies

## Abstract

Colorectal cancer (CRC) is a major cause of cancer-related mortality worldwide, with a significant impact on public health. Current treatment options include surgery, chemotherapy, radiotherapy, molecular-targeted therapy, and immunotherapy. Despite advancements in these therapeutic modalities, resistance remains a significant challenge, often leading to treatment failure, poor progression-free survival, and cancer recurrence. Mechanisms of resistance in CRC are multifaceted, involving genetic mutations, epigenetic alterations, tumor heterogeneity, and the tumor microenvironment. Understanding these mechanisms at the molecular level is crucial for identifying novel therapeutic targets and developing strategies to overcome resistance. This review provides an overview of the diverse mechanisms driving drug resistance in sporadic CRC and discusses strategies currently under investigation to counteract this resistance. Several promising strategies are being explored, including targeting drug transport, key signaling pathways, DNA damage response, cell death pathways, epigenetic modifications, cancer stem cells, and the tumor microenvironment. The integration of emerging therapeutic approaches that target resistance mechanisms aims to enhance the efficacy of current CRC treatments and improve patient outcomes.

## 1. Introduction

### 1.1. Colorectal Cancer Incidence and Risk Factors

Colorectal cancer (CRC) is a disease that includes both colon cancer and rectal cancer, often grouped together because they have many common features. The incidence rates for CRC in the United States from 2016 to 2020 were 35.3 per 100,000 people, with 40.7 in 100,000 for men and 30.6 per 100,000 for women [1]. While CRC incidence rates have gone down overall since their peak in 1985, the prevalence of CRC has been rising in younger adults (20–49 years) for decades [2]. The median age of diagnosis for CRC is 67; however, younger patients tend to present with a more advanced-stage disease compared to older patients [3]. In the United States and worldwide, CRC is the third most common cancer diagnosed in both men and women, and the second leading cause of cancer-related deaths when numbers for men and women are combined [1,4]. CRC is particularly deadly because it can grow undetected without causing symptoms for many years and may not cause significant health problems until the tumor is large or at an advanced stage and may have spread to other parts of the body.

There are many factors that can increase the risk for CRC, including both non-modifiable biological risk factors and modifiable lifestyle risk factors [5]. Biological or genetic factors include sex, age, race and ethnicity, prior colon diseases, family history of CRC, and health conditions such as inflammatory bowel disease or type 2 diabetes mellitus. Lifestyle factors include diet (high in processed or red meat and low in fruits and vegetables), physical inactivity, being overweight or obese, smoking, and alcohol use. While the overall decline in CRC incidence in the United States over the past 35 years can be partly attributed to reductions in smoking and increased use of nonsteroidal anti-inflammatory drugs, the increased prevalence of CRC screening by colonoscopy is primarily credited for reducing CRC incidence in people aged 50 and older [2]. Like other solid tumor types, CRC that is detected at an early stage when the tumor is small and has not spread is more likely to be treated successfully.

### 1.2. Colorectal Cancer Pathogenesis

CRC occurs when cells in the colon or rectum grow and divide uncontrollably and can form anywhere along the length of the large intestine. Colon cancers account for ~70% of CRC cases, while the remaining ~30% are rectal cancers, and both types of cancer can spread throughout the body if left untreated [2]. Within the colon, most tumors develop in the distal colon (~70%), which includes the descending colon, sigmoid colon, and distal one-third of the transverse colon [6]. The location of a tumor in the proximal colon (right side), which includes the cecum, ascending colon, and proximal two-thirds of the transverse colon, or the distal colon (left side), can affect detection, prognosis, and treatment response [6]. Most sporadic CRCs start as a benign growth of tissue on the mucous layer of the colon or rectum called a polyp, which can become cancerous over time if left untreated [7]. A series of genetic mutations in the polyp’s cells can lead to the development of CRC carcinogenesis, a process that generally takes 10–15 years.

The histological type of CRC is determined by the type of tumor cells in the biopsy, and can be classified as adenocarcinoma, which is the most common type of CRC (~95% of all CRC cases), or one of the rare types, such as neuroendocrine tumors (also called carcinoid tumors) or colorectal primary lymphomas. CRC is staged based on whether the tumor has spread beyond the primary site in the colon or the rectum, and if so, where in the body it has spread (e.g., lymph nodes, nearby tissues, or distant organs) [5]. CRC stages range from 0 (non-invasive or “in situ”) to 4 or metastatic (spread to distant organs). The most common site for CRC metastasis is the liver, followed by the lungs and bones [8].

The clinical presentation of CRC ranges from asymptomatic to severe illness. CRC can be detected early, before it starts to cause symptoms through CRC screening modalities such as colonoscopy and stool-based tests [9]. For advanced CRC cases, symptoms can include rectal bleeding, blood in stool, change in bowel habits, abdominal pain, abdominal mass, bowel obstruction, constipation, diarrhea, iron-deficiency anemia, weight loss, and fatigue [7,8]. Right-sided CRC is associated with younger age, and common signs and symptoms include diarrhea, bowel obstruction, and constipation, whereas left-sided CRC is more common in older patients, who often present with rectal bleeding and bowel obstruction [10,11]. If the cancer has metastasized, patients may present with other symptoms depending on where in the body it has spread, such as jaundice or swelling (liver), breathing issues (lung), or pain and fractures (bone).

### 1.3. Genetic Mutations and Associated Molecular Pathways

CRC is characterized by genetic alterations, including gene mutations that target tumor suppressor genes, oncogenes, and DNA repair mechanisms. CRC is commonly associated with mutations in key driver genes including *APC*, *KRAS*, *BRAF*, *TP53*, *SMAD4*, and *PIK3CA* [12]. Almost all sporadic CRCs have mutations in the Wnt/β-catenin signaling pathway, most being partial or complete loss-of-function of the tumor suppressor APC protein (*APC* mutated in ~80% of CRCs), which leads to unrestricted activation of Wnt signaling and downstream expression of genes important for cell proliferation, stemness, and survival [12,13]. In addition, the epidermal growth factor receptor (EGFR)/RAS/MAPK signaling pathway is often constitutively activated, either through activation of signaling proteins RAS (*KRAS* mutated in ~40% of CRCs) or RAF (*BRAF* mutated in 10–15% of CRCs), which stimulates cell proliferation [13,14,15]. Loss-of-function mutations in the tumor suppressor genes *TP53* (mutated in 50–60% of CRCs) and *SMAD4* (mutated in ~15% of CRCs) also occur frequently [13,16]. Loss of p53 causes defects in DNA damage response and DNA repair, leading to genomic instability [12], whereas loss of SMAD4 impacts cell proliferation and differentiation pathways and is associated with CRC progression [17]. Mutations in *PIK3CA* (mutated in ~20% of CRCs) result in activation of the PI3K/AKT/mTOR signaling pathway, which promotes cell proliferation and survival [13,18].

Sequential mutations in driver genes—known as the adenoma–carcinoma sequence—are thought to occur at the transitions from normal epithelium to pre-malignant tumor to malignant tumor [19]. Initial mutations in the Wnt/β-catenin pathway (*APC*, *CTTNB1*) and the RAS/MAPK pathway (*KRAS*, *BRAF*) drive adenoma development. The accumulation of additional mutations in p53 (*TP53*), transforming growth factor-β (TGF-β; *SMAD4*, *TGFBR2*), and PI3K/AKT (*PIK3CA*, *PTEN*) signaling pathways are required for progression to carcinoma. Invasive and metastatic transformation likely involves additional alterations, such as chromosomal instability and other genetic and epigenetic modifications [20]. Although most CRC tumors harbor common driver gene mutations, they can vary greatly in their biology, thus indicating poor genotype–phenotype correlation in CRC.

### 1.4. Epigenetic Alterations and Associated Molecular Pathways

In addition to genetic changes, epigenetic alterations have been identified as important contributors to CRC development and progression [21]. Epigenetic modifications can alter gene expression without permanently changing the underlying DNA sequence [22] and are influenced by aging and environmental and lifestyle factors. CRC presents different types of epigenetic alterations, including DNA methylation, histone modifications, and non-coding RNA (ncRNA)-associated changes [21,23]. Alterations in DNA methylation have been identified in genes that are frequently mutated in CRC and in genes involved in DNA repair and the Wnt/β-catenin pathway [24]. Aberrant DNA hypermethylation occurs in the promoter regions of many tumor suppressor genes, including *APC*, *CDKN2A*, *MLH1*, and *CDH1* [25]. Furthermore, DNA hypomethylation at regulatory and/or coding regions has been linked to the activation of proto-oncogenes [21]. The most common histone modifications in the context of CRC are acetylation and methylation, which can lead to the activation or repression of gene expression, depending on the degree of histone acetylation or the residue that is methylated [21]. Aberrant histone acetylation and methylation at specific marks have been identified in CRC, along with deregulation of several enzymes that modify histones [26]. For example, altered expression of histone-modifying enzymes is associated with inactivation of the CRC tumor suppressor genes *CDH1*, *CDKN1A*, *NDRG1*, and *PPARG* [26]. Additionally, ncRNA species such as microRNAs (miRNAs) and long non-coding RNAs (lncRNAs) also have roles as epigenetic regulators in CRC and are implicated in many cancer-related pathways, including the Wnt/β-catenin, RAS/MAPK, p53, TGF-β, and PI3K/AKT signaling pathways [21].

### 1.5. Colorectal Cancer Subtypes

From a molecular perspective, CRC has traditionally been classified into subtypes based on the two major mechanisms of genomic instability, key genetic mutations, and epigenetic alterations. This genome-based molecular subtyping depends on features of chromosomal instability (CIN), microsatellite instability (MSI), and CpG island methylator phenotype (CIMP) [27]. CIN refers to an increased rate of acquisition of gross numerical or structural alterations in chromosomes (duplications or deletions), resulting in activation of proto-oncogenes, inactivation of tumor suppression genes, and loss of heterozygosity. CIN is a hallmark of the classical pathway of CRC tumorigenesis, intrinsically associated with *APC*, *KRAS*, *TP53,* and *SMAD4* mutations, and accounts for ~85% of sporadic CRCs [12]. MSI occurs when there are changes in the number of microsatellites, which are short sequences of DNA that are repeated throughout the genome and is caused by deficient DNA mismatch repair (dMMR). In CRC, MSI is intrinsically associated with mutations in MMR genes, such as *MLH1*, *MSH2*, *MSH6,* and *PMS2*, or can be caused by biallelic hypermethylation of the *MLH1* promoter [21]. MSI accounts for ~15% of sporadic CRCs [12]. CIMP is characterized by hypermethylation of CpG islands at the promoters of several genes, including tumor suppressor genes, thus leading to transcriptional inactivation. CIMP contributes to both the MSI and CIN pathways and is observed in ~20% of sporadic CRCs [12]. In CRC, CIMP is intrinsically associated with hypermethylation of the *MLH1* promoter and mutations in *BRAF* [21]. While useful, the classification of CRC into these categories has come with certain challenges, including overlap between subtypes, substantial heterogeneity within subtypes, and limited prognostic value.

To resolve inconsistencies among CRC subtype classifications, an international consortium formed across expert groups and developed a gene expression-based classification system based on six different published CRC subtyping platforms to systematically assign CRC consensus molecular subtypes (CMS) [28]. The study looked at gene expression data from >3000 CRC patients from around the world and additional molecular data where available on gene mutations, genomic aberrations, and immune activation. The authors determined that 87% of CRC samples analyzed could be assigned to one of four consensus molecular subtypes with distinguishing features (CMS1–4), while the other 13% had mixed features [28]. CMS1 (MSI immune, 14%) tumors are hypermutated, microsatellite unstable, and have strong immune activation, with frequent occurrence of *BRAF* mutations. CMS2 (canonical, 37%) tumors are epithelial and have strong upregulation of Wnt and MYC signaling pathways and more frequent somatic copy number alterations (SCNA). CMS3 (metabolic, 13%) tumors are epithelial with evident metabolic deregulation and overrepresentation of *KRAS* mutations. CMS4 (mesenchymal, 23%) tumors have prominent TGF-β activation, stromal infiltration and angiogenesis, and more frequent SCNA. Furthermore, the authors determined that subtype correlated with patient outcome. CMS2 was associated with better survival rate after relapse, whereas CMS1 was associated with worse survival rate after relapse, and CMS4 had worse relapse-free and overall survival. The CMS classification system has improved our understanding of CRC heterogeneity and prognosis and is a potential basis for clinical stratification and future subtype-based targeted treatment.

## 2. Therapeutic Approaches for Colorectal Cancer

### 2.1. Standard Therapies

**Figure 1:**
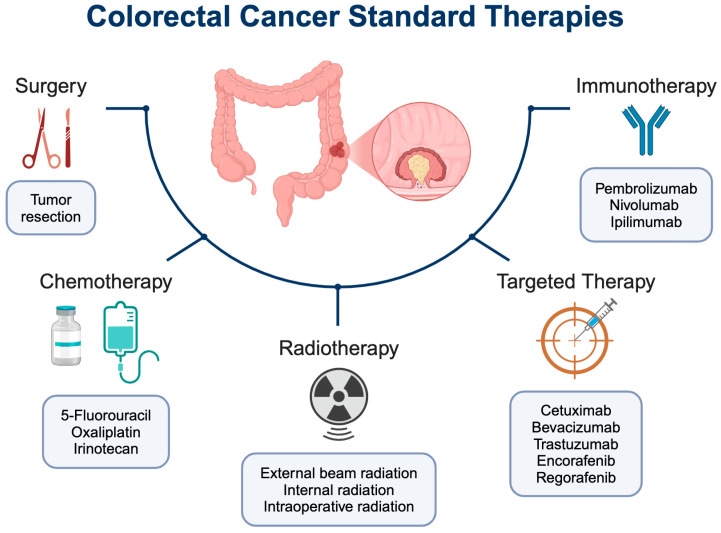
Therapeutic approaches for colorectal cancer (CRC). The standard therapies for CRC are surgery, chemotherapy, radiotherapy, targeted therapy, and immunotherapy. Combinations of standard therapies are often used to treat CRC. Illustration created in https://www.biorender.com/, accessed on 11 February 2025.

Standard therapies for CRC include surgery, chemotherapy, radiotherapy, molecular-targeted therapy, and immunotherapy (Figure 1). CRC therapies are often given in combination with one another in order to maximize the response. Treatment recommendations are based on multiple factors, including cancer stage, location, how likely the cancer is to spread, whether it is newly diagnosed or has recurred after previous treatment(s), and the patient’s overall health. In addition, CRC subtype and the presence of certain biomarkers inform treatment recommendations, helping to tailor therapies to individual patients [29]. Surgery is the primary therapy for most CRC and is often the first-line treatment to remove cancerous tissue, especially in the early stages of the disease [8]. The standard-of-care of newly diagnosed colon cancer is surgery plus adjuvant chemotherapy, although neoadjuvant chemotherapy may also be used to shrink a large tumor prior to surgery. For rectal cancer, the standard-of-care is surgery plus chemoradiotherapy.

**Figure 2:**
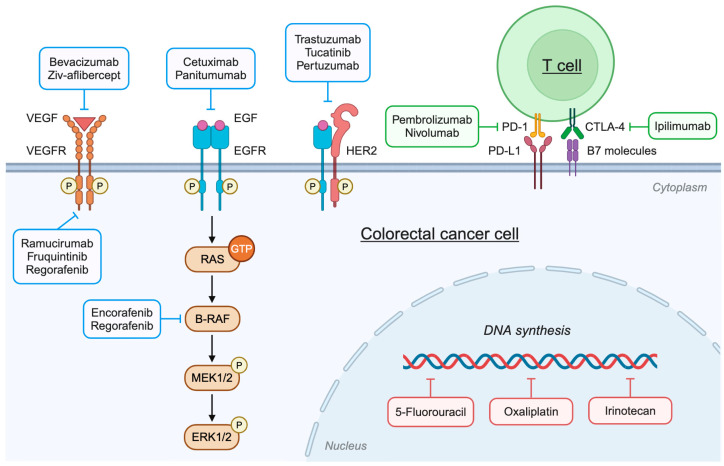
Molecular targets of current anti-CRC drugs. The drugs indicated are FDA-approved for CRC treatment. Chemotherapy drugs are shown in red outline boxes, targeted therapy drugs are shown in blue outline boxes, and immunotherapy agents are shown in green outline boxes. Anti-VEGF/VEGFR therapies also target endothelial cells, and anti-CTLA-4 therapies also target dendritic cells. Illustration created in https://www.biorender.com/.

Chemotherapy uses chemicals to kill or slow the growth of rapidly dividing cancer cells. The most common chemotherapy drugs for CRC are 5-Fluorouracil (5-FU), oxaliplatin, and irinotecan (Figure 2) [8]. 5-FU is an antimetabolite antineoplastic agent and a key chemotherapy drug for CRC. It is a pyrimidine analog that blocks DNA synthesis, leading to cell death [30,31]. 5-FU is usually administered in combination with folinic acid (leucovorin), which enhances the cytotoxic effects of 5-FU. Alternatively, capecitabine, which is an orally available 5-FU prodrug, may be taken. Oxaliplatin is a platinum-based antineoplastic drug used to treat advanced CRC. It is an alkylating agent that damages DNA and inhibits DNA synthesis [30,31]. Irinotecan is an antineoplastic drug used to treat metastatic CRC. It is a topoisomerase I inhibitor that blocks DNA replication and transcription [30,31]. Chemotherapy is usually given as a combination of two or three chemotherapy drugs, such as FOLFOX, FOLFIRI, FOLFIRINOX, or CAPOX (acronyms for the different components in each combination).

Radiotherapy, or radiation therapy, uses high-energy radiation to kill cancer cells and may be given before, during, and/or after surgery. Radiotherapy can be administered as external beam radiation therapy (EBRT), internal radiation therapy (brachytherapy), or intraoperative radiation therapy (IORT) [32]. It is used to shrink a tumor before surgery, kill any cancer cells remaining after surgery, and/or decrease the chances of recurrence. Radiotherapy is used more often to treat rectal cancer than colon cancer [33].

### 2.2. Targeted Therapies

Molecular-targeted therapy uses drugs to target specific molecules on cancer cells, which can help stop the growth, progression, and spread of cancer. Several targeted therapies are used to treat advanced or metastatic CRC, the selection of which depends on the absence or presence of genetic biomarkers [29].

The two most common molecular targets in CRC are vascular endothelial growth factor (VEGF) and epidermal growth factor receptor (EGFR) (Figure 2) [34]. Anti-VEGF therapies, which include VEGF inhibitors, such as bevacizumab and ziv-aflibercept, and VEGF receptor (VEGFR) inhibitors, such as ramucirumab and fruquintinib, block the formation of tumor blood vessels, or angiogenesis [35]. Anti-EGFR therapies, such as EGFR tyrosine kinase inhibitors cetuximab and panitumumab, block or slow down the growth of tumor cells. This type of targeted therapy is only effective in the absence of mutations in the *RAS* and *BRAF* genes.

Less common targeted therapies for CRC include HER2 inhibitors (e.g., trastuzumab, tucatinib, pertuzumab, and trastuzumab deruxtecan) for *HER2*-amplified (HER2-positive) advanced CRC that is *KRAS*-WT and *BRAF*-WT, and BRAF inhibitors (e.g., encorafenib) for metastatic CRC with *BRAF-V600E* mutation, used in combination with EGFR inhibitor cetuximab (Figure 2) [14,34]. Also, multi-kinase inhibitors (e.g., regorafenib) may be used to treat metastatic CRC that has progressed after treatment with other drugs and for which there are no other treatment options [34]. Additional rare, targeted therapies used to treat advanced CRC with specific gene fusions are tropomyosin receptor kinase inhibitors (e.g., larotrectinib) for *NTRK*-fusion, and rearranged during transfection inhibitors (e.g., selpercatinib) for *RET*-fusion [36].

### 2.3. Immunotherapies

Immunotherapies are medicines that help the body’s immune system recognize and destroy cancer cells. In CRC, immunotherapy is a new therapeutic approach that can be used to treat advanced or metastatic CRC in some patients, depending on certain biomarkers. Immune checkpoint inhibitors (ICIs) are a class of immunotherapy that is often used to treat MSI High (MSI-H) or dMMR tumors [37]. This subset of CRC exhibits high immunogenicity due to increased mutational burden, inducing high immune cell infiltration, particularly from tumor-infiltrating lymphocytes [37,38]. The most common ICIs are PD-1 inhibitors (e.g., pembrolizumab, nivolumab) and CTLA-4 inhibitors (e.g., ipilimumab), which activate T cells to induce an antitumor response from the immune system (Figure 2) [38].

## 3. Mechanisms of Drug Resistance

**Figure 3:**
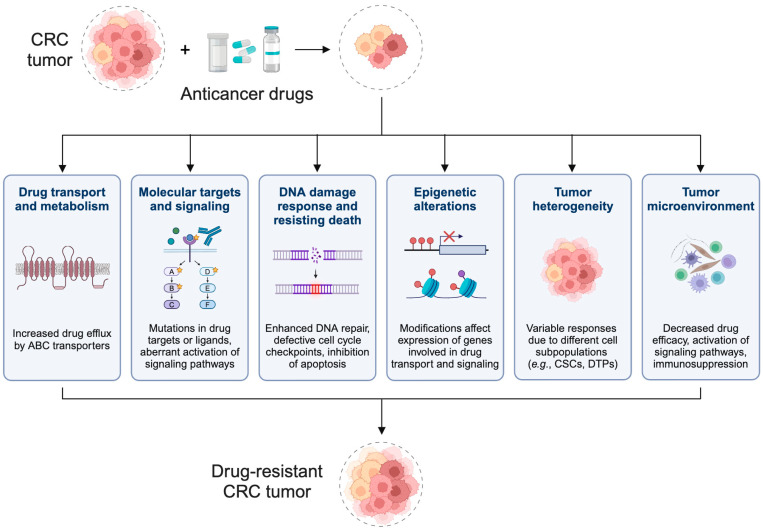
Mechanisms of drug resistance in CRC. Various molecular and cellular mechanisms contribute to drug resistance in sporadic CRC. ABC, ATP-binding cassette; CSCs, cancer stem cells; DTPs, drug-tolerant persisters. Illustration created in https://www.biorender.com/.

One of the greatest challenges to CRC treatment is the high frequency of drug resistance, which undermines the success of CRC therapies. While most CRC patients initially respond to therapy, the overall response rate to first-line treatments can vary widely, depending on the patient tumor characteristics and treatment regimen [39]. Furthermore, cancer recurrence occurs in 30–40% of patients who completed CRC treatment, with most recurrences happening in the first 2 to 3 years after initial treatment [40]. While overall survival rates for individuals with advanced CRC has increased in recent decades, drug resistance has been reported to develop in nearly all CRC patients [5]. The development of drug resistance varies, but it can occur quickly, within weeks of starting treatment, or may take months or years. Resistance mechanisms in CRC are diverse and include both intrinsic (or innate) and acquired forms (Figure 3) [41]. Intrinsic resistance is often linked to genetic mutations present before treatment, which can render certain therapies ineffective, or may be a feature of some tumor cell subpopulations. Acquired resistance, on the other hand, develops during treatment and can involve secondary mutations, alterations in drug transport or signaling pathways, upregulation of DNA repair, and various other molecular mechanisms. Furthermore, the tumor microenvironment (TME) plays a significant role in mediating resistance. The development of multidrug resistance (MDR), which is the acquisition of resistance to multiple, structurally unrelated chemotherapy drugs, is a major obstacle in the treatment of cancers, including CRC [42]. A comprehensive understanding of the underlying mechanisms of drug resistance in CRC is critical for developing effective therapeutic strategies to overcome resistance and improve patient outcomes.

### 3.1. Drug Transport and Metabolism

Drug resistance in CRC is influenced by alterations in drug transport and metabolism. Reduced drug influx is a common mechanism of resistance, often resulting from decreased expression of transporters responsible for drug uptake, such as membrane solute carrier (SLC) family proteins [43]. Conversely, CRC cells can increase drug efflux by overexpression of ATP-binding cassette (ABC) transporters, such as P-glycoprotein (PGP, also known as multidrug resistance protein 1 or MDR1), breast cancer resistance protein (BCRP), and MDR-associated proteins (MRP1, MRP2) [41]. ABC transporters actively pump a wide range of anticancer drugs out of cancer cells, thereby reducing their intracellular concentrations [44]. Overexpression of ABC transporters is thought to be the main cause of MDR in cancer cells [45]. In addition, changes in drug metabolism contribute to resistance, with CRC cells increasing the inactivation or degradation of drugs through upregulated expression of enzymes like glutathione S-transferases (GSTs), uridine 5′-diphospho-glucuronosyltransferases (UGTs), and cytochrome P450 (CYP) enzymes [46,47]. Changes in drug transport and metabolism lead to decreased efficacy of the administered therapies, primarily affecting chemotherapy drugs, such as 5-FU, oxaliplatin, and irinotecan.

### 3.2. Molecular Targets and Signaling Pathways

**Figure 4:**
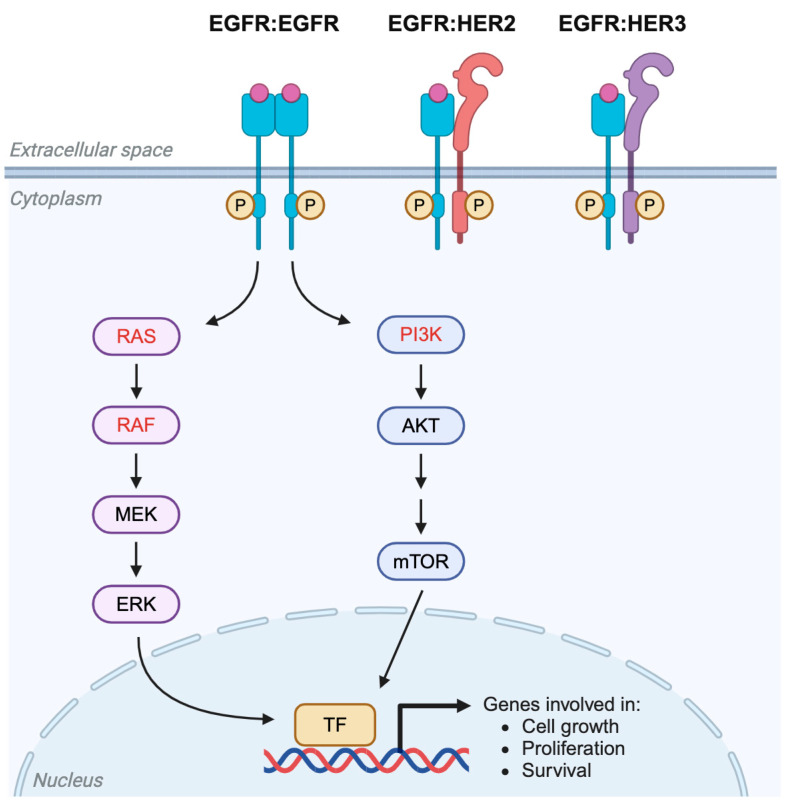
EGFR signaling pathways. Upon binding of ligands (e.g., EGF), EGFR forms homodimers and initiates intracellular signaling to two downstream pathways, MAPK and PI3K. These signaling pathways lead to transcriptional activation and expression of genes involved in cell growth, proliferation, and signaling. Ligand-bound EGFR can also form heterodimers with other EGFR family receptors such as HER2 or HER3. Proteins in red font are often constitutively activated due to mutation in CRC. TF, transcription factor. Illustration created in https://www.biorender.com/.

Drug resistance in CRC is linked to alterations in molecular targets and signaling pathways. In CRC tumors that initially respond to therapy, secondary mutations in drug targets and/or their ligands can render therapies ineffective by preventing drug binding or activation, thus leading to acquired resistance. For instance, increased expression of thymidylate synthase or reduced expression of the topoisomerase in response to therapy can induce resistance to chemotherapy drugs 5-FU or irinotecan, respectively, and potentially contribute to MDR [41]. In addition, mutations in the extracellular domain of EGFR or upregulation of alternative EGFR ligands amphiregulin and epiregulin are likely mechanisms of anti-EGFR therapy resistance in *KRAS-WT* CRC patients [41]. Furthermore, alterations in downstream signaling pathways can sustain proliferative signals despite the inhibition of upstream targets. Common activating mutations in *RAS* or *BRAF* genes are well-known predictors of resistance to anti-EGFR therapies like cetuximab, which work by blocking the EGF/RAS/RAF/ERK signaling pathway (Figure 4) [48]. Moreover, since EGFR signals to two downstream pathways, there is increasing evidence that aberrant activation of the PI3K/AKT/mTOR pathway can also mediate anti-EGFR therapy resistance [41]. Activation of alternative receptors can also drive resistance by independently activating the inhibited pathway. For instance, *HER2* gene amplification or activating mutations, or *MET* gene amplification can lead to persistent RAS/MAPK signaling, thereby reducing the efficacy of anti-EGFR therapies [41]. Finally, the chronic activation of oncogenic/bypass signaling pathways, like Wnt/β-catenin, TGF-β, Janus kinase (JAK), and signal transducer and activator of transcription (STAT), maintain the growth and survival of CRC cells, thereby circumventing the effects of various [41]. Changes in molecular targets and related signaling pathways can affect treatment response to all types of therapy but are most relevant to resistance to targeted therapies.

### 3.3. DNA Damage Response and Cell Death

Most chemotherapy drugs used to treat CRC work by either interfering with DNA replication or by damaging DNA, which then activates cell death pathways. Altered DNA damage response (DDR) in CRC cells can lead to therapy resistance through various mechanisms. CRC cells often have enhanced DNA damage repair capacity due to upregulation of alternative pathways and can fix DNA damage induced by chemotherapy or radiotherapy [49]. Defective cell cycle checkpoints allow CRC cells to bypass damage-induced arrest and proceed through the cell cycle with damaged DNA [50]. Activation of survival pathways can promote cell survival and proliferation in the presence of DNA-damaging agents [49]. Defective DDR can also cause changes in the TME by releasing inflammatory cytokines, which create an immunosuppressive state that helps CRC cells evade immune surveillance, thereby reducing the effectiveness of immunotherapies [51]. Furthermore, the inherent genomic instability of CRC cells leads to the accumulation of mutations potentially resulting in drug-resistant clones that can expand under treatment [51].

Resisting cell death is an important hallmark of cancer cells and a key contributor to therapy resistance in CRC. Apoptosis is a non-lytic form of programmed cell death (type I) and is the primary mechanism by which therapies use to eliminate cancer cells [52]. Defects in apoptosis are common in CRC and are mainly due to inactivating mutations in *TP53*, and increased expression of anti-apoptotic proteins (e.g., BCL-2, BCL-XL) or decreased expression of pro-apoptotic proteins (e.g., BAX, BIM) [47]. Inhibition of apoptosis pathways impedes CRC cell death and confers resistance to various therapies, including chemotherapy, radiotherapy, and some targeted therapies. Furthermore, defective apoptotic pathways can contribute to MDR [45].

Autophagy is a distinct form of programmed cell death (type II) that involves degradation of cellular organelles and recycling the components [53]. In the context of cancer, autophagy can either inhibit or promote tumorigenesis, depending on the cancer type, stage, and genetic background. Normally, autophagy plays a tumor suppressive role by preventing the accumulation of misfolded proteins, damaged organelles, and reactive oxygen species (ROS) [54]. However, in established tumors, autophagy is often used by cancer cells as a survival mechanism in response to stress conditions like nutrient deprivation, low oxygen (hypoxia), and chemotherapy [53]. Autophagy can help CRC cells survive by degrading damaged organelles and proteins and providing nutrients for energy metabolism. Enhanced autophagy pathways are associated with CRC resistance to chemotherapy [41].

### 3.4. Epigenetic Alterations

Alterations in the CRC epigenome can play a significant role in contributing to resistance against conventional drugs, such as 5-FU, oxaliplatin, irinotecan, and cetuximab. Epigenetic alterations can affect the expression of genes involved in drug transport and several signaling pathways in CRC cells, such as cell proliferation, survival, apoptosis, and DNA repair [23,55]. Key molecular mechanisms include DNA methylation, histone modifications, and ncRNA-associated changes [23]. For example, hypermethylation of the tumor suppressor gene *MLH1* can lead to resistance against 5-FU by preventing the recognition and repair of DNA damage [56]. Alterations in histone modifications can regulate gene expression and chromatin structure, further contributing to resistance against chemotherapy drugs [57,58,59]. Moreover, alterations in histone acetylation and methylation can lead to changes in *KRAS* expression, and H3K9 and H3K27 methylation can repress *EGFR* transcription, thus leading to resistance against cetuximab [55]. Dysregulation of ncRNAs, such as miRNAs and lncRNAs, can lead to altered drug sensitivity and resistance in CRC cells [60]. For instance, certain miRNAs can regulate expression of target genes that are involved in drug response pathways, e.g., *TP53*, *BCL2* (apoptosis) and *KRAS*, *PTEN* (cell proliferation and survival) [61]. In addition, different ncRNAs have been shown to contribute to resistance against 5-FU, oxaliplatin, and anti-EGFR therapies by various mechanisms [62,63,64]. Epigenetic alterations in CRC cells can also mediate therapy resistance through interactions with components of the TME, including immune cells, stromal cells, and extracellular matrix (ECM) proteins [65]. Furthermore, epigenetic alterations can modulate cells in the TME, such as immune cells and fibroblasts, thereby rewiring the TME and suppressing the immune response, which drives tumorigenesis and contributes to immunotherapy resistance [23,66].

### 3.5. Tumor Heterogeneity and Cell Subpopulations

CRC is characterized by a high degree of molecular heterogeneity among tumors, which accounts for differences in disease progression and response to therapy. Inter-tumoral heterogeneity describes the existence of different molecular profiles between tumors from different patients, whereas intra-tumoral heterogeneity refers to the presence of different molecular profiles within a single tumor due to tumor cell subpopulations that have distinct genetic, epigenetic, and phenotypic profiles [67]. Intra-tumoral heterogeneity poses a major challenge for CRC treatment, as different cell subpopulations may respond variably or even resist therapy and promote recurrence [23]. In addition, CRC cellular and phenotypic plasticity can also contribute to tumor heterogeneity and therapy resistance or evasion.

Cancer stem cells (CSCs) are a phenotypically and functionally heterogeneous subpopulation of cells within tumors that have distinct stem-like properties. Like normal adult stem cells, CSCs can self-renew indefinitely and differentiate into non-stem cell progeny (multipotent), thereby generating the majority of cancer cells in a tumor [68]. Colorectal CSCs are highly tumorigenic and have been defined by specific cell surface markers, including CD133, CD44, CD166, CD24, CD26, LGR5, and EpCAM [69]. CSCs also play an important role in metastasis and are resistant to various therapies, which makes them a key factor contributing to cancer recurrence [68]. CSCs have numerous inherent characteristics that promote therapy resistance. While conventional therapies target rapidly proliferating and more differentiated cancer cells, CSCs are slow-growing or quiescent and poorly differentiated [70]. Colorectal CSCs exhibit aberrant activation of signaling pathways, such as Wnt/β-catenin, Notch, Hedgehog, Hippo/YAP, and PI3K/AKT, which promote cell growth, survival, and self-renewal [69]. They also have a higher expression of ABC transporters, enhanced DNA repair mechanisms, and improved immune evasion [71]. Epigenetic modifications also contribute to drug resistance by promoting maintenance and self-renewal of colorectal CSC through various signaling pathways [66]. In addition, CSCs can adapt in response to chemotherapy and radiotherapy by altering cell cycle checkpoints, by inducing cell cycle arrest (reversible quiescent G_0_ state), or by efficient scavenging of ROS [70]. Moreover, colorectal CSCs exhibit significant cellular plasticity in that they can transition between stem like and differentiated states, which further increases tumor heterogeneity and escape from therapeutic targeting [72].

Epithelial–mesenchymal transition is a cellular process that involves switching from an epithelial to a mesenchymal cell phenotype and occurs during normal physiological processes, such as development and wound healing [73]. During EMT, cells downregulate expression of the epithelial cell adhesion protein E-cadherin and upregulate expression of EMT transcription factors, including SNAIL, ZEB, and TWIST families, leading to a loss of cell–cell adhesion and polarity and a gain in cell motility [74]. In cancer, EMT plays an important role in tumor invasion, metastasis, and resistance to various therapies [75]. There are several known molecular mechanisms by which EMT promotes therapy resistance in CRC. These include altered drug transport, e.g., overexpression of drug efflux pumps like ABC transporters, activation of cell survival signaling pathways such as PI3K/AKT, and deregulation of apoptotic pathways leading to resistance to apoptosis [76]. In addition, EMT-induced CRC cells interact differently with their microenvironment, leading to enhanced ECM remodeling and immune evasion [77]. EMT is also associated with a CSC-like phenotype in CRC tumors, while colorectal CSCs are reported to have enhanced EMT characteristics [71].

Drug-tolerant persisters (DTPs) are a small subpopulation of cells within tumors that can survive anticancer drug treatment by entering a transient quiescent or slow cycling state (dormancy) [78]. The DTP state is reversible, as cells resume growth and proliferation upon removal of drug treatment, giving rise to a tumor cell population that remains sensitive to therapy [79]. Notably, DTPs do not have permanent genetic mutations that confer resistance [79]. Instead, they rely on different non-genetic mechanisms of adaptation to evade therapy-induced cell death and promote survival, including epigenetic remodeling, transcriptional or translational rewiring, altered metabolism, phenotypic plasticity, and microenvironmental interactions [80]. For instance, CRC DTPs were found to induce an embryonic survival phenotype (diapause) that activates autophagy, in response to the chemotherapy drug irinotecan [81]. While biomarkers to identify DTPs are lacking, findings from various studies in different cancers suggest that DTPs can either arise from selection of a pre-existing cell population or emerge de novo during drug exposure, depending on the experimental model and treatment used [80]. DTPs share some characteristics with CSCs; however, DTPs have distinctive features such as the ability to enter transient quiescence and display phenotypic plasticity, which have crucial roles in contributing to drug resistance and tumor relapse [80].

### 3.6. Tumor Microenvironment

The TME surrounding CRC cells is different from the normal tissue microenvironment and can reduce treatment efficacy and lead to therapy resistance through various mechanisms. The TME is composed of both non-cellular and cellular components. Non-cellular components include the ECM—composed of proteins, proteoglycans, and glycoproteins—cytokines, growth factors, and enzymes like proteases [82]. Cellular components of the TME include stromal cells (fibroblasts, endothelial cells, pericytes, mesenchymal stem cells) and immune cells (T cells, B cells, macrophages, neutrophils, and dendritic cells). Compared to the microenvironment of healthy tissue, the TME has augmented deposition of proteins such as collagen and fibronectin, high cytokine levels, increased proportion of fibroblasts and immune cells, and is immunosuppressive [82,83].

The mechanisms by which the TME is known to promote therapy resistance in CRC are numerous and diverse. Dense ECM, abnormal vasculature, and an acidified environment all contribute to decreased drug penetration [83]. Increased levels of cytokines and growth factors in the TME can activate cell proliferation and survival signaling pathways in CRC cells (e.g., MAPK and PI3K/AKT) [84]. Abnormal or poorly organized tumor vasculature also results in hypoxia, which stabilizes hypoxia-inducible factor (HIF) transcription factors that activate signaling pathways regulating survival, angiogenesis, and metabolism [85]. Hypoxia can induce cell cycle arrest, upregulate anti-apoptotic proteins, and increase expression of drug resistance genes, such as drug efflux pumps. Furthermore, hypoxia promotes self-renewal by activation of Wnt and Notch signaling pathways and embryonic stem cell transcription factors, and it modulates differentiation of tumor cells and tumor-associated stromal cells [86,87,88]. Additionally, both increased matrix stiffness and hypoxia can induce EMT of CRC cells [89,90].

Different cell populations in the TME mediate resistance to CRC therapies. Cancer-associated fibroblasts (CAFs) support CRC growth, proliferation, invasion, and metastasis and promote angiogenesis and immune remodeling [91]. Relative to normal fibroblasts, CAFs have increased production of ECM, cytokines, and growth factors, including PDGF, HGF, VEGF, and CXCL12 [71]. CAFs also secrete factors and extracellular vesicles that activate signaling pathways in CRC cells promoting stemness and EMT, thereby contributing to drug resistance [91]. Moreover, CAFs secrete pro-inflammatory factors that alter the sensitivity of CRC cells and protect them from chemotherapy drugs [92].

The TME is immunosuppressive, thus rendering the immune system less effective in killing cancer cells. Myeloid-derived suppressor cells (MDSCs), a heterogeneous group of immune cells from the myeloid lineage, are a major component of the immunosuppressive TME [93]. MDSCs participate in immunosuppression by inhibiting T and NK cell function, which interferes with the antitumor immune response [41]. Cytokines secreted by CRC cells recruit MDSCs to the tumor site and confer resistance to immunotherapies like ICIs [41]. The TME is also characterized by chronic inflammation, which is linked to tumor cell proliferation, survival, and metastasis [94]. Inflammatory cells such as macrophages and neutrophils can cause immunosuppression in the TME. In CRC, tumor-associated macrophages (TAMs), which are polarized towards an M2-like phenotype, have been demonstrated to enhance tumor proliferation, invasion, and metastasis, stimulate tumor-related angiogenesis, and inhibit the antitumor immune response [95]. TAMs also secrete TGF-β, which recruits and activates Tregs that participate in immunosuppression [96]. CSCs secrete inflammatory factors and cytokines, such as IL-6, IL-4, VEGF, CCL2, and TGF-β, that recruit macrophages to the TME and activate them to become TAMs [97].

## 4. Strategies to Overcome Drug Resistance

**Figure 5:**
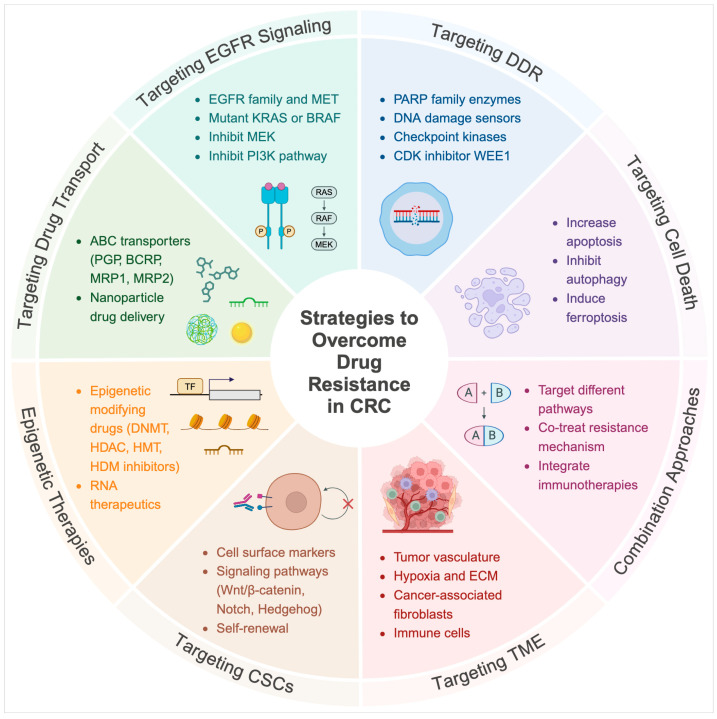
Strategies to overcome drug resistance in CRC. Several promising strategies are being explored to overcome drug resistance in sporadic CRC. CDK, cyclin-dependent kinase; CSCs, cancer stem cells; DDR, DNA damage response; DNMT, DNA methyltransferase; ECM, extracellular matrix; HDAC, histone deacetylase; HDM, histone demethylase; HMT, histone methyltransferase; TME, tumor microenvironment. Illustration created in https://www.biorender.com/.

CRC therapy resistance is a significant challenge, but recent advances in existing and novel therapeutic approaches offer promise for overcoming drug resistance. Strategies to overcome resistance may involve blocking compensatory mechanisms or alternative pathways used by cancer cells to escape standard therapies, thereby restoring drug sensitivity (Figure 5) [49]. Otherwise, it could be achieved by targeting specific cancer cell populations responsible for tumor initiation and metastasis that remain viable after conventional therapies like chemotherapy and radiotherapy. Alternatively, or in combination with one of the above strategies, modifying the TME to enhance drug efficacy or increase the immune response could be very beneficial. This section describes current and emerging strategies to overcome CRC drug resistance and improve patient outcomes.

### 4.1. Targeting ABC Membrane Transporters

Targeting drug efflux pumps like ABC transporters (PGP, BCRP, MRP1, and MRP2) would increase the intracellular concentration of chemotherapy drugs, thereby improving their effectiveness in tumor cells.

Studies are being conducted to develop new approaches to block efflux pump activity or downregulate transporter expression, design drugs that are not ABC transporter substrates, or use nanotechnology to evade efflux mechanisms [49]. While various ABC transporter inhibitors have been shown to sensitize drug-resistant colon cancer cells to chemotherapy drugs in experimental models [98,99,100,101], the development of PGP as a therapeutic target for cancer in general has not been successful clinically [42]. The utility of pharmacological inhibitors of ABC transporters may be complicated due to compensatory mechanisms cancer cells use to overcome inhibition, such as increased mRNA expression, as well as potential adverse side effects on normal cells [44]. In addition, the expression of ABC transporters varies considerably across patient tumors, and the expression of multiple ABC transporters in a single tumor is common [42]. ABC transporter inhibitors may therefore be most effective in a subset of CRC patients whose cancer cells overexpress a specific ABC transporter. Notably, the research in this field has recently shifted to focusing on natural products and subsequent structural modifications to develop novel ABC transporter inhibitors with good safety and efficacy profiles [47].

Another method being investigated to target ABC transporters is downregulating their expression by RNA interference-based therapeutics, such as miRNAs. Recent research has revealed substantial posttranscriptional gene regulation of ABC transporters by miRNAs, underscoring the potential utilization of novel bioengineered miRNA agents to downregulate ABC transporter expression and affect intracellular drug accumulation [101]. In various cancers, including CRC, miRNAs that target ABC transporters have been reported to modulate the chemosensitivity of cancer cells [102,103]. Moreover, several studies have demonstrated that using miRNA mimics to increase the levels of specific miRNAs, such as miR-133b, miR-142-3p, miR-297, and miR-522, can increase the chemosensitivity of drug-resistant CRC cells [104,105,106,107]. Thus, using miRNAs that target ABC transporters as therapeutic agents could help to overcome CRC chemoresistance.

An alternative approach to targeting transporters is the emerging field of nanomedicine, which involves the use of nanoparticles (NPs) to deliver drugs directly into cancer cells, thereby bypassing transporters and limiting damage to healthy cells. Research is ongoing to examine the utility of biocompatible NPs, such as metal-based NPs, carbon-based NPs, polymeric NPs, and liposomes, as drug delivery tools to treat CRC [108,109]. For instance, gold nanoparticles have been used to effectively deliver various anti-CRC therapies to tumor cells, including chemotherapy drugs and anti-EGFR inhibitors, resulting in enhanced anticancer drug efficacy [110,111,112]. Polymeric polylactide-co-glycolic acid (PLGA) NPs were also shown to increase the cytotoxicity of chemotherapy drugs, such as 5-FU and irinotecan, in CRC cell lines [113,114]. Furthermore, EGF-targeted 5-FU-loaded PLGA NPs were developed to selectively target CRC cells expressing high levels of EGFR and showed higher rates of cytotoxicity and apoptosis and lower rates of tumor growth compared to non-targeted or non-NP treatments [115]. Similarly, folic acid-targeted 5-FU-loaded liposomes were demonstrated to have greater efficacy in CRC cells in vitro and in vivo compared to liposomal 5-FU or free drug [116,117]. More recently, several nanocarriers have been created to specifically address MDR in CRC therapy by co-delivering chemotherapy drugs and ABC transporter inhibitors [109].

### 4.2. Targeting EGFR and Alternative Pathways

The EGFR/RAS/RAF/MEK/ERK pathway is a key regulator of CRC progression and metastasis, making EGFR an important molecular target for CRC therapy (Figure 4). However, there are multiple mechanisms of resistance to anti-EGFR therapy (both inherent and acquired), including mutations in EGFR extracellular domain, amplification of other receptors such as *HER2* or *MET*, activating mutations in *RAS* or *BRAF*, and aberrant activation of PI3K signaling [41]. Targeting molecules involved in these resistance mechanisms would help to overcome resistance to anti-EGFR monoclonal antibody (mAb) therapies like cetuximab and panitumumab. Studies are currently underway to develop new mAbs targeting EGFR family receptors or alternative receptors [29]. For instance, Sym004 is a mixture of two mAbs that bind distinct epitopes of EGFR and may have the potential to overcome acquired resistance due to EGFR mutations that inhibit the binding of conventional anti-EGFR antibodies to EGFR [118]. Also in development are novel therapies involving mAbs that bind other EGFR family receptors, such as HER2 and HER3, which can form heterodimers with EGFR to initiate downstream signaling [29]. Antibodies targeting HER2, such as trastuzumab and pertuzumab, have shown promising activity in patients with HER2-positive metastatic CRC [119,120]. Additionally, the combination of anti-MET and anti-EGFR therapies may provide an effective therapeutic option for CRC patients with acquired resistance to anti-EGFR antibody therapy due to MET receptor amplification [29].

Activating mutations in the RAS/RAF/MEK/ERK signaling pathway downstream of EGFR generally confer resistance to anti-EGFR therapies; however, recent developments have led to new approaches for therapeutically targeting CRC with *RAS* or *BRAF* mutations [29]. Although RAS was previously thought to be undruggable, pharmacological inhibition of mutant KRAS is now possible. For example, sotorasib and adagrasib are selective, small molecule inhibitors of KRAS-G12C that are approved for non-small cell lung cancer and pancreatic cancer, which have the *KRAS-G12C* mutation. Recently, adagrasib was also approved, in combination with cetuximab, for advanced CRC with *KRAS-G12C* that has progressed after treatment with chemotherapy. There are additional drugs currently in development for targeting G12C, G12V, or other mutations (e.g., G12D), as well as non-selective, multi-KRAS mutant inhibitors (active against G12C, G12D, G12V, and G12X) [121]. Other approaches under investigation to target mutant KRAS in CRC include perturbing the interaction between KRAS and the plasma membrane, and the combined inhibition of downstream pathways [122]. For cancers with activating *BRAF* mutations, various BRAF inhibitors are now available, such as vemurafenib, dabrafenib, and encorafenib, that selectively and competitively inhibit mutant BRAF and interfere with downstream MAPK signaling [14]. BRAF inhibitors are widely used to treat melanoma with *BRAF-V600* mutations, and encorafenib is also approved, in combination with cetuximab, for metastatic CRC with *BRAF-V600E* mutation, which is the predominant *BRAF* mutation in CRC [29]. However, acquired resistance to BRAF-targeted therapies in *BRAF*-mutated CRC is a significant challenge, but it may potentially be overcome using combinations of anti-BRAF and anti-EGFR therapies with inhibitors of MEK, Wnt, or cyclin-dependent kinases CDK4/6 [29].

Another approach being investigated for targeting resistance to anti-EGFR therapy involves inhibiting downstream signaling pathways, MAPK and PI3K/AKT/mTOR. For instance, a combination of anti-EGFR therapy with a MEK inhibitor, such as selumetinib or pimasertib, could potentially limit acquired resistance to anti-EGFR antibody therapy from activating mutations in *RAS* or *BRAF* [29]. However, while MEK inhibitors have shown clinical efficacy in several types of *KRAS*-mutated cancers, preclinical studies in CRC revealed that treatment with MEK inhibitor alone was not very effective in *KRAS*-mutated CRC cells due to different mechanisms of acquired resistance [123,124,125]. Activating mutations in *PIK3CA* can also contribute to resistance to anti-EGFR therapy in *KRAS-WT* CRC patients by persistent activation of the PI3K/AKT/mTOR signaling pathway [126]. Various drugs targeting this pathway are under investigation for CRC treatment, including pan-PI3K inhibitors, isoform-specific PI3K inhibitors, dual PI3K-mTOR inhibitors, Akt inhibitors, and mTOR inhibitors [126]. While PI3K pathway inhibitors as a monotherapy have not shown significant antitumor effects in treating CRC, their utility in combination therapy is actively being studied. For instance, compensatory signaling is known to occur between RAS/RAF/MEK/ERK and PI3K/AKT/mTOR pathways, which provides strong scientific rationale for combining MEK and PI3K inhibitors. Indeed, results from several preclinical and clinical studies indicate that dual pharmacological targeting of MAPK and PI3K pathways may be an effective strategy to inhibit the growth of CRC cells, regardless of *KRAS* or *PI3K* mutational status [127,128,129,130,131]. However, while the combination of MAPK and PI3K inhibitors holds therapeutic potential, there are associated toxicities that may limit the doses that can be administered to CRC patients. Thus, new or modified approaches are being explored to improve the tolerability of these combinations.

### 4.3. Targeting DNA Damage Response

Dysregulated DDR and enhanced DNA repair pathways enable CRC cells to resist therapy-induced DNA damage and cell death. Therefore, inhibiting DDR would help to overcome resistance and improve the efficacy of treatments such as chemotherapy and radiotherapy. Research is in progress to determine the landscape of DDR alterations in different cancer types, including CRC, and exploit DDR defects as novel therapeutic targets for anticancer treatment [132]. Since cells use several pathways to recognize and repair different types of DNA damage, DDR inhibitors targeting different DDR pathways are under investigation in cancer cells, alone or in combination with other therapies [51].

Poly (ADP-ribose) polymerase (PARP) inhibitors are a class of drugs that target PARP family enzymes, which play a crucial role in facilitating the repair of DNA single-strand breaks. By inhibiting PARP, these drugs block the repair of single-strand breaks, leading to the accumulation of double-strand breaks and cell death [49]. PARP inhibitors such as olaparib can be effective in cancers with defects in homologous recombination repair and are currently approved for several cancers harboring *BRCA* mutations, including breast, ovarian, pancreatic, and prostate cancers [51]. Various preclinical studies have demonstrated PARP inhibitors to also be effective in CRC cells; however, the sensitivity may depend on the status of certain genes involved in DDR [132]. For example, a subset of CRC cell lines enriched for *KRAS* or *BRAF* mutations were found to be highly sensitive to the PARP inhibitor olaparib and also displayed functional deficiency in homologous recombination [133]. Additionally, PARP inhibitors have also shown efficacy when used in combination with DNA-damaging agents such as chemotherapy and radiotherapy [50,134]. For instance, PARP inhibition was shown to sensitize CRC cells to irinotecan, which was dependent upon homologous recombination deficiency [135]. Furthermore, PARP inhibitors enhanced the anticancer activity of chemoradiation in CRC cell lines in vitro and in vivo [136,137,138].

Other DDR proteins that may act as potential therapeutic targets include DNA damage sensor kinases DNA-PK and ATM/ATR, cell cycle checkpoint kinases CHK1/CHK2, and cyclin-dependent kinase inhibitor WEE1. Currently, there are several DDR inhibitors targeting ATM, ATR, DNA-PK, CHK1, or WEE1 being investigated for treating various cancers, including CRC [51]. For instance, a selective ATM inhibitor was shown to enhance the sensitivity of CRC cell lines to irinotecan in vitro and confer drug sensitivity to CRC patient-derived xenografts that were resistant to irinotecan monotherapy [139]. WEE1 is a tyrosine kinase that regulates the G2/M checkpoint of the cell cycle by preventing cells with DNA damage from entering mitosis, and so inhibiting WEE1 could sensitize tumors to DNA-damaging therapies [132]. A WEE1 inhibitor was recently reported to exert anticancer effects in CRC cells, particularly in those with *TP53* mutations [140]. This is likely because cancer cells with mutant p53 have a defective G1/S checkpoint and are more reliant on the G2/M checkpoint for DNA repair, suggesting a potential therapeutic vulnerability. Additionally, various studies indicate that DDR inhibitors can sensitize CRC cells to the anticancer effects of chemotherapy and radiotherapy [50]. Overall, a better understanding of DDR alterations in CRC is needed to help identify patient subsets who would be the most likely to benefit clinically from PARP or other DDR inhibitors.

### 4.4. Targeting Cell Death Pathways

Targeting cell death pathways would help to overcome therapy resistance due to dysregulation of these and other related pathways. Research is ongoing to develop strategies to increase apoptosis, inhibit autophagy, and induce an alternative cell death pathway called ferroptosis [52,53,141].

Targeting the apoptotic core machinery, including BCL-2 family proteins, inhibitors of apoptosis proteins (IAPs), death receptors, and caspases, can promote cancer cell death and reduce resistance [52]. For example, drugs targeting the BCL-2 family of proteins, such as BH3 mimetics, have been investigated for their ability to induce apoptosis in CRC cells [142,143]. Furthermore, the IAP antagonist TL32711 was demonstrated to sensitize CRC cell lines to 5-FU- or oxaliplatin-induced cell death [144]. In addition, death receptor-targeted therapies (e.g., DR4/DR5 agonist antibodies) were shown to enhance the antitumor activity of chemotherapy drugs (5-FU, irinotecan) in CRC xenograft models [145,146,147]. Notably, several existing drugs have been repurposed as potential anticancer agents, including some non-steroidal anti-inflammatory drugs (NSAIDs), statins (cholesterol lowering drugs), and metformin (anti-diabetic drug), which were shown to augment apoptosis and overcome chemoresistance in CRC cells [148].

Autophagy is often used by cancer cells to survive the stress of chemotherapy, thus promoting therapeutic resistance. Targeting key molecules involved in autophagy (e.g., Beclin-1, ATG5, ATG7) can modulate autophagy to enhance cancer cell cytotoxicity and reduce resistance [53]. Several studies demonstrated that inhibiting autophagy by treatment with chloroquine or hydroxychloroquine, which are repurposed anti-malarial drugs, increased the sensitivity of CRC cells to 5-FU, oxaliplatin, and chemoradiotherapy [149,150,151]. Moreover, inhibiting autophagy could also target therapy-resistant CSCs and DTPs, both of which often have upregulated autophagy pathways [80,152].

Ferroptosis is a unique form of regulated cell death that involves iron accumulation and lipid peroxidation. Factors that induce ferroptosis can affect glutathione peroxidase (GPX) activity through different pathways, resulting in decreased cellular antioxidant capacity and accumulation of lipid ROS, eventually leading to oxidative damage and cell death [153]. The use of ferroptosis inducers in CRC cells can promote iron-dependent lipid peroxidation, leading to cell death [141]. For instance, the ferroptosis inducer RSL3 (small molecule inhibitor of GPX4) initiated ROS accumulation and cell death in multiple CRC cell lines [154]. Furthermore, inducing ferroptosis could also be used to target CRC DTPs, as GPX4 and ferrous iron were upregulated in 5-FU-tolerant persister cells, which were sensitive RSL3-induced ferroptosis in xenograft tumor models [155]. Additionally, erastin, a selective inhibitor of SLC7A11 (a cystine–glutamate antiporter also known as xCT) exhibited a synergistic effect in inducing ferroptosis in CRC cells when combined with oxaliplatin [156]. Other ferroptosis-based strategies could involve interventions in iron metabolism and lipid peroxidation [46].

### 4.5. Epigenetic Therapies

The goal of epigenetic therapies is to reverse alterations in gene expression that contribute to drug resistance. Studies are ongoing to develop approaches that prevent or overcome CRC resistance mechanisms using epigenetic modifying drugs or RNA therapeutics [21,66]. While it is unclear if there is clinical benefit from epigenetic drugs used alone, various epigenetic drugs are being evaluated in combination with chemotherapeutic and other antitumor agents for cancer therapy. In theory, epigenetic drugs can be used to reprogram tumor cells to re-sensitize them to conventional therapies, which could potentially lower doses of cytotoxic drugs and improve tolerability [21]. Since epigenetic drugs modulate various components of the TME, they are also being tested in combination with immunotherapy (e.g., ICIs) for their ability to increase tumor antigen expression, processing, and presentation, thereby aiming to reverse immune evasion [157].

There are several different classes of drugs that inhibit enzymes responsible for epigenetic modifications, including DNA methyltransferases (DNMTs), histone deacetylases (HDACs), histone methyltransferases (HMTs), and histone demethylases (HDMs) [21,66]. DNMT inhibitors target aberrant DNA hypermethylation in cancer cells, leading to re-expression of silenced tumor suppressor genes. However, excessive demethylation by DNMT inhibitors can also disrupt normal gene expression patterns and cause toxicity [158]. DNMT inhibitors include cytidine analogs that incorporate into DNA (e.g., azacitidine and decitabine) and non-nucleoside inhibitors that directly interact with DNMTs [158]. Several studies have shown the combination of DNMT inhibitors and standard chemotherapy drugs to be effective in CRC cells. For instance, multiple DNMT inhibitors exhibited additive or synergistic effects when combined with 5-FU, oxaliplatin or irinotecan in CRC cell lines [159,160,161]. Moreover, DNMT inhibitors were demonstrated to restore 5-FU sensitivity to 5-FU-resistant CRC cells in mouse xenograft tumor models [162,163].

HDAC inhibitors prevent the removal of acetyl groups from histones, leading to a more open or decondensed chromatin structure and changes in gene expression. They work as anticancer drugs by inducing cell cycle arrest, differentiation, and cell death, and by reducing angiogenesis and modulating the immune response; however, the mechanisms of their effects can differ depending on the individual HDAC inhibitor and its dose and on the type of cancer [164]. Numerous studies have demonstrated HDAC inhibitors to be effective in CRC cells when used in combination with chemotherapy drugs such as 5-FU, oxaliplatin, and irinotecan [21,66]. For instance, pan-HDAC inhibitors exhibited synergistic effects when combined with 5-FU in CRC cell lines in vitro or with FOLFOX or FOLFIRI in CRC carcinomas in a preclinical study, thus showing a potential role for HDAC inhibitors as chemosensitizers [165,166]. Mechanistically, various studies suggest that inhibiting HDACs can overcome resistance to chemotherapy-induced DNA damage in CRC cells by different molecular mechanisms, such as downregulating thymidylate synthase gene expression or modifying chromatin condensation, and subsequently inducing cell death [58,59,165]. Additionally, a selective inhibitor of HDAC6 was reported to enhance the chemotherapeutic effects of 5-FU or oxaliplatin in CRC cells [167,168].

Histone methylation is a dynamic process regulated by HMTs and HDMs that can have a positive or negative effect on gene transcription, depending on the site of the modification [66]. HMT and HDM inhibitors are being investigated for their potential to reduce CRC cell growth and overcome drug resistance. For instance, targeting enzymes like Enhancer of Zeste Homologue 2 (EZH2; subunit of HMT that trimethylates H3K27) has shown promising inhibitory effects on proliferation, migration, invasion, and colony/sphere formation of CRC cell lines [169,170]. Additionally, inhibiting EZH2 reduced tumor growth and CSC self-renewal and increased chemosensitivity to 5-FU in CRC patient-derived xenograft models [171]. Targeting EZH2 may also be an effective strategy to overcome oxaliplatin resistance, since EZH2 was shown to promote oxaliplatin resistance in CRC cells, while EZH2 inhibitor suppressed tumor growth and sensitized CRC cells to oxaliplatin [172,173]. On the other hand, inhibiting Lysine Demethylase 1A (KDM1A; demethylase for H3K4 and H3K9) suppressed CRC cell proliferation through downregulation of the Wnt/β-catenin pathway [174]. Furthermore, targeting KDM4 (demethylase for H3K9 and H3K36) may be used to overcome cetuximab resistance in CRC with high *EGFR* gene copy number, since inhibiting KDM4 reduced *EGFR* amplifications in CRC cells [175].

Another epigenetics-based approach to overcoming resistance mechanisms and helping restore drug sensitivity is modulating ncRNAs. miRNAs have potential as therapeutic targets either by replacing downregulated tumor suppressor miRNAs (e.g., using miRNA mimics) or by inhibiting upregulated oncogenic miRNAs (e.g., using small interfering RNAs or antisense oligonucleotides) [21]. For instance, inhibition of miR-520g, which increases resistance to 5-FU by reducing p21 expression, was shown to sensitize colon cancer cells to 5-FU-induced apoptosis [176]. Similarly, inhibition of miR-19a or miR-454-3p, which are upregulated in oxaliplatin-resistant cells and target *PTEN* gene expression, partially reversed oxaliplatin resistance in CRC cell lines [177,178]. On the other hand, miR-483-3p is downregulated in oxaliplatin-resistant CRC cells, and miR-483-3p mimics restored oxaliplatin responsiveness via increased apoptosis [179]. In addition, lncRNAs are also being investigated as potential therapeutic targets for overcoming CRC chemoresistance [66]. However, an important limitation of the use of ncRNAs is the challenge of passing hydrated liposomal delivery vectors across hydrophobic membranes of tumor cells, underpinning the need for molecular modifications or the design of vehicles to aid specific delivery [21].

### 4.6. Targeting Colorectal Cancer Stem Cells

Targeting CSCs would enhance the efficacy of anti-CRC therapies, reduce metastasis, and lower recurrence. Several approaches are being explored to specifically target CSCs to reduce therapy resistance and improve patient outcomes [69,71,72,180]. One of the key approaches in targeting CSCs involves the use of cell surface markers, such as CD133, EpCAM, EGFR, and LGR5, which are overexpressed in CSCs compared to normal stem cells. These markers have facilitated the development of bio-targeted therapies that can selectively bind to and eradicate CSCs [180]. For example, LGR5-targeted antibody–drug conjugates were demonstrated to induce cytotoxicity in LGR5-high gastrointestinal cancer cells and decrease tumor size and proliferation in colon cancer xenograft models [181,182]. However, a potential limitation of this type of therapy is that CSCs share molecular similarities with normal stem cells; therefore, more specific targeting approaches are necessary.

Bispecific antibodies (BsAbs) are an emerging class of therapeutics that contain two different antigen-binding domains within one molecule and can been used to target colorectal CSCs [183]. For instance, MCLA-158 (petosemtamab), a BsAb targeting both EGFR and LGR5, exhibited strong growth inhibition of patient-derived CRC organoids and orthotopic xenograft tumor models, including *KRAS* mutant CRCs resistant to cetuximab [184]. Importantly, MCLA-158 selectively triggered EGFR degradation in LGR5+ colorectal CSCs but showed minimal toxicity toward normal LGR5+ colon stem cells. Also being developed for CRC are bispecific T-cell engagers (BiTEs), which are a subtype of BsAbs that simultaneously bind a tumor-associated antigen on cancer cells and CD3 on T cells, thereby activating an immune response [185]. For example, EpCAM/CD3 BiTEs eliminated CSCs in colon cancer cell lines and primary CSCs isolated from colon cancer patient samples and blocked CRC xenograft tumor growth [186,187]. Additionally, EGFR-specific BiTEs were shown to lyse *KRAS*- and *BRAF*-mutated CRC cell lines in vitro and inhibit the growth of CRC xenograft tumors, while cetuximab was ineffective [188]. Another emerging type of immunotherapy is oncolytic virus therapy, which uses oncolytic viruses to selectively kill tumor cells while sparing healthy cells [185]. Oncolytic viruses work by directly killing cancer cells and by stimulating an antitumor immune response. Researchers have engineered oncolytic viruses to target receptors on tumor cells, including CSC markers. For instance, an oncolytic adenovirus targeting CD133 was shown to selectively infect and kill CD133+ colorectal CSCs and inhibit growth of CRC xenograft tumors [189].

A second key approach for targeting colorectal CSCs involves the suppression of specific signaling pathways that are critical for CSC maintenance and proliferation [69,71,72]. The Wnt/β-catenin signaling pathway is a prime candidate to target because it is crucial for maintaining CSC properties of self-renewal and proliferation, and it is aberrantly activated in most sporadic CRCs. Wnt signaling activity can be modulated by targeting various molecules along the pathway from Wnt receptors at the plasma membrane (Frizzled and LRP5/6) to downstream transcription factors in the nucleus (β-catenin and TCF/LEF) [72]. In CRC cells, Wnt pathway inhibition has been demonstrated to inhibit cell proliferation, reduce tumor-initiating cell numbers, and impair tumor growth in CRC xenograft models [190,191,192,193]. There is also a substantial amount of preclinical data supporting the use of Wnt pathway inhibitors in various types of cancer including CRC, and some of these agents are in different stages of clinical research [194]. For instance, niclosamide (Frizzled receptor inhibitor) and ICG-001 (β-catenin/TCF inhibitor) are being studied in clinical trials for CRC and other solid tumors. However, it is important to note that in the context of CRC, the potential efficacy of a specific agent targeting the Wnt pathway will depend on whether and how the pathway is activated in tumor cells. For example, agents that target Wnt receptors or Wnt ligand secretion (e.g., porcupine inhibitor LGK974) can downregulate pathway signaling in Wnt ligand-dependent tumors but are not expected to be effective in CRCs that harbor *APC* or *CTTNB1* mutations. Additionally, recently developed technologies are currently being explored to inhibit Wnt signaling, such as proteolysis-targeting chimeras and molecular glue degraders, antibody–drug conjugates, and antisense oligonucleotides [195].

The Notch signaling pathway plays a significant role in CSC self-renewal and cell fate decisions, and dysregulated Notch signaling contributes to CRC progression, chemoresistance, and CSC heterogeneity [196]. Current therapeutic approaches to target Notch signaling include γ-secretase inhibitors (GSIs), mAbs, and natural products [183]. GSIs, which inhibit Notch signaling by preventing the proteolytic cleavage of Notch receptors, have been shown to reduce the self-renewal of colorectal CSCs and promote their differentiation [69]. Neutralizing mAbs targeting Notch receptors or ligands, such as delta ligand-4 (DLL4), have also been developed to block Notch signaling and were reported to reduce colorectal CSC self-renewal and tumor growth in vivo [196]. Other methods currently being investigated to inhibit the Notch pathway include using small molecule inhibitors against the Notch-dependent transcription factor HES-1, or the matrix metalloproteinase ADAM17, which is involved in the cleavage and activation of Notch receptors [72]. While inhibiting Notch signaling is an appealing strategy for CRC treatment, it does present certain challenges, as Notch1 was previously shown to counteract Wnt/β-catenin signaling in mouse colon cancer cells [197]. Additionally, the development of Notch pathway modulators that selectively target cancer cells while sparing healthy cells is imperative [69].

The Hedgehog (Hh) signaling pathway, which is critical for organ development and tissue homeostasis, also plays a role in tumorigenesis and CSC maintenance. Aberrant activation of Hh signaling has been implicated in the pathogenesis of several types of cancer; however, its role in CRC is controversial and may depend in part on the CRC subtype and stage [198]. The Hh signaling pathway involves Hh ligands binding to the transmembrane receptor Patched, activation of the transmembrane protein Smoothened (SMO) to initiate downstream signaling, and subsequent activation of GLI transcription factors [71]. The most effective way to inhibit Hh signaling is by using SMO inhibitors, such as cyclopamine, vismodegib, and sonidegib. While numerous preclinical studies have shown that SMO inhibition can limit therapy resistance, tumor recurrence, and metastasis in various malignancies, the results in CRC are less clear [71]. This may be because in the gastrointestinal tract, Hh signaling is conducted by two Hh homologs, Sonic Hedgehog (SHH) and Indian Hedgehog (IHH), that have different roles in gut development and homeostasis [199]. Moreover, most studies in CRC support a stimulatory role for SHH and an inhibitory role for IHH in colorectal tumorigenesis [198]. Nevertheless, inhibiting Hh signaling in CRC cells has been shown to be effective in reducing cell viability and expression of CSC markers and may also enhance chemosensitivity at least in some cellular contexts [200,201,202]. Thus, further studies are needed to determine the efficacy of using Hh inhibitors in combination with standard therapies and to identify CRC subtypes that are most likely to benefit from Hh inhibition based on Hh pathway activation [69].

Another potentially valuable approach involves targeting the self-renewal capacity of colorectal CSCs to irreversibly impair their tumorigenic potential and drive differentiation. For instance, treatment of patient-derived CRC xenografts with a small molecule inhibitor of BMI-1 (a subunit of the Polycomb Repressive Complex 1 that functions as an epigenetic chromatin modifier) resulted in loss of colorectal CSCs and long-term impairment of tumor growth [203]. Differentiation therapy uses agents such as retinoic acid to induce CSCs to differentiate into less tumorigenic cells, making them unable to sustain tumor growth and potentially more susceptible to other therapies [204]. For example, treatment of colon cancer cell lines with the compound all-trans retinoic acid inhibited cell proliferation, decreased CSCs, induced differentiation, and enhanced the efficacy of 5-FU in vitro [205,206]. Other agents, including bone morphogenetic protein 4 (BMP4) and some HDAC inhibitors, have also been shown to promote differentiation of colorectal CSCs and increase their response to chemotherapy [207,208,209]. Furthermore, disrupting the bivalent epigenetic state through EZH2 inhibition was shown to both reduce self-renewal of CRC patient-derived CSCs and upregulate expression of differentiation genes, resulting in increased sensitivity to 5-FU in vivo [171].

Additionally, repurposed drugs like metformin and NSAIDs, as well as natural products such as curcumin, salvianolic acid, and vitamin C are being studied for their ability to target colorectal CSC self-renewal, proliferation, apoptosis, and ROS pathways and help overcome therapy resistance [148,210].

### 4.7. Targeting Tumor Microenvironment

The TME plays an important role in CRC progression and therapy resistance. Targeting the TME would help to overcome resistance by enhancing drug efficacy, disrupting signaling pathways that promote tumor growth and survival, and/or activating antitumor immunity.

Normalizing tumor vasculature can improve delivery of chemotherapy drugs and increase oxygenation, thereby making tumor cells more susceptible to treatments. Anti-angiogenic therapies, which inhibit the growth of new blood vessels, are currently given in combination with chemotherapy to treat patients with advanced or metastatic CRC [211]. Different types of anti-angiogenic agents are used to block the VEGF-VEGFR signaling axis, including mAbs (bevacizumab, ramucirumab), soluble decoy receptors (ziv-aflibercept), and small molecule multi-kinase inhibitors (regorafenib) [35]. However, since anti-angiogenic therapies lead to only modest increases in survival for CRC patients, there is a need for biomarkers for patient stratification, integration of novel combinations of anti-angiogenic drugs with immunotherapy, and better preclinical models of metastatic CRC [211].

Reducing hypoxia can improve the efficacy of standard therapies like chemotherapy and radiotherapy. Drugs that block the activity of HIF transcription factors (HIF inhibitors) are currently being investigated as potential therapeutics to target mechanisms that promote survival and therapy resistance in hypoxic tumor cells [85]. Moreover, HIF-1 inhibitors have been shown to enhance the sensitivity of CRC cells to the cytotoxic effects of chemotherapy drugs like 5-FU and oxaliplatin [212,213,214]. Additionally, as hypoxia is known to contribute to immunosuppression and ICI resistance, targeting hypoxia may also enhance the effectiveness of immunotherapies [215].

An alternative approach to targeting tumor hypoxia involves the use of specific drugs designed to exploit tumor hypoxia. Hypoxia-activated prodrugs (HAPs), which are also called bioreductive drugs, become active only in low-oxygen environments, thus selectively targeting hypoxic regions of solid tumors [49]. For instance, the HAP evofosfamide (also known as TH-302) was reported to enhance the anticancer activity of chemotherapy drugs in mouse xenograft models of various cancer types, including CRC [216]. Furthermore, evofosfamide was shown to effectively reduce CRC tumor growth and CSC numbers when administered sequentially after 5-FU treatment [217].

Targeting ECM components like collagen can reduce ECM density and stiffness, thereby improving drug penetration and efficacy in CRC tumors. Modulating ECM stiffness can also disrupt signaling pathways that promote EMT. Several therapeutic approaches have been developed that aim to alleviate excessive collagen deposition in the TME of solid tumors by altering the synthesis, degradation, or crosslinking of collagen [218]. For example, inhibiting lysyl oxidase (LOX) family enzymes, which are involved in the crosslinking of ECM proteins collagen and elastin, can reduce matrix stiffness and improve drug delivery to tumor cells [219]. In CRC, high *LOXL2* expression is associated with poor prognosis, increased recurrence, and contributes to 5-FU chemoresistance [220]. Various drugs targeting LOXL2 are currently under investigation for CRC treatment, including β-aminopropionitrile, simtuzumab, and PXS compounds [220]. In addition, several other ECM components, such as fibronectin, hyaluronan, TGF-β, integrins involved in mechanotransduction, and other sensors of matrix stiffness, are being studied as potential therapeutic targets in various cancers, including CRC [218]. However, agents that degrade and/or deconstruct the ECM should be used cautiously, as they may lead to the release of factors that promote tumor progression and induce metastasis [83].

Nano-targeted drug delivery systems are also being explored as a novel means to deliver anticancer drugs directly to the TME to enhance their efficacy. Specific targeting strategies are being developed to deliver drugs to the colorectal TME, which is characterized by high levels of ROS, acidic pH, and elevated temperature [221]. For example, a synthesized dual drug-loaded pH/thermo-responsive hydrogel, which is a hydrophilic three-dimensional porous network, induced greater apoptotic cell death in CRC cells compared to the single drug-loaded carriers or free drugs [222].

Another strategy to modify the TME to help overcome therapy resistance involves targeting non-tumor cells that promote tumorigenesis and/or suppress immune responses. Stromal cells within the TME are attractive therapeutic targets because they are genetically stable with reduced risk of developing resistance [223]. Inhibiting tumor–stromal cell interactions could disrupt signaling pathways that promote CRC growth, survival, and EMT. CAFs are a significant component of the CRC tumor stroma and secrete ECM proteins like collagen and other factors that promote resistance to various treatments, including chemotherapy, anti-EGFR therapy, and immunotherapy [41]. Different therapeutic approaches are currently being investigated for their potential to specifically target CAFs, including a variety of inhibitors, agonists, and neutralizing antibodies that interfere with different molecules or signaling pathways that contribute to the supportive role of CAFs in the TME [224]. For instance, in CRC, fibroblast activation protein was identified as a strong candidate for antibody conjugate therapy since it was found to be highly expressed in most CRC tissues and absent in >90% of adjacent normal tissues (colon and liver) [225]. Additionally, studies are being conducted to design specific EMT inhibitors that will enhance the efficacy of conventional therapies and reduce metastasis [49].

Targeting immune cells in the TME can reverse immunosuppression and stimulate antitumor immunity. Research is ongoing to develop new and improved strategies to harness the immune system to overcome therapy resistance in cancer [41,49,180]. Different types of immunotherapies have recently emerged as potential powerful therapeutics for treating various cancers, including CRC. Checkpoint inhibitors are designed to inhibit pathways upregulated in cancer cells that are used to avoid immune detection and elimination, such as the PD-1/PD-L1 pathway [49]. For example, ICIs like pembrolizumab and nivolumab block the PD-1 receptor, which activates T cells and allows them to better recognize and destroy tumor cells, even those that have become resistant to other treatments [49]. In CRC, ICIs are currently used to treat a special subset of patients with advanced or metastatic disease, that is MSI-H or dMMR; however, further studies are being undertaken to explore new approaches to enhance the overall effectiveness of ICIs in treating CRC.

In addition to ICIs, there are various emerging immunotherapies that have shown promising results in preclinical models and/or other cancer types and may be used in the future to treat CRC and help overcome resistance. This includes BiTEs and oncolytic viruses (discussed above), antitumor vaccines, and different types of adoptive cell transfer, such as chimeric antigen receptor (CAR) T cell therapy, tumor-infiltrating lymphocyte therapy, and T cell receptor therapy [37,38,226]. Adoptive cell transfer therapies generally involve extracting a patient’s immune cells, manipulating them in the lab, then reintroducing the immune cells back into the patient to target and eliminate cancer cells [49]. In CAR-T cell therapy, T cells are collected and genetically modified to express CARs that recognize specific antigens on cancer cells, then infused back into the patient to help the immune system identify and kill cancer cells [185]. This type of immunotherapy can tackle drug resistance by targeting cancer-specific or cancer-associated antigens that are not affected by conventional treatments [49]. Several antigens being evaluated as candidates for anti-CRC CAR-T cell therapy have shown efficacy in CRC preclinical models, including carcinoembryonic antigen and guanylyl cyclase 2C [227,228]. Furthermore, T cells can be engineered to express CARs that selectively target CSC markers, leading to improved recognition and destruction of CSCs, which are commonly resistant to T cell therapy [180]. For instance, CAR-T cells currently under development for targeting colorectal CSCs include CD133-, EpCAM-, or CD166-directed CAR-T cells [229,230,231,232].

Targeting inflammation in the TME can also reduce cancer progression, enhance immune responses, and overcome therapy resistance. Research is ongoing to develop TAM-directed strategies that either prevent macrophages from entering the TME or regulate their repolarization. One potential approach is to modulate the colorectal TME by inhibiting signaling molecules or pathways that promote the recruitment of monocytes and macrophages to the tumor site, such as the CCL2/CCR2 and CSF-1/CSF-1R pathways [41,95]. Alternatively, TAMs can be reprogrammed to polarize towards a pro-inflammatory anti-tumorigenic M1 phenotype. For instance, the small molecule immunotherapy drug tasquinimod induced TAM phenotype switching from M2 to M1 and inhibited tumor growth and vascularization in a mouse colon carcinoma model [233]. Additional preclinical studies showed that repolarizing TAMs enhanced T-cell-mediated anti-tumor immune response and improved the efficacy of ICIs in CRC tumors [234,235].

### 4.8. Combination Approaches

Combination therapy approaches can enhance anticancer efficacy and help overcome or delay the development of resistance. The standard-of-care for CRC treatment has evolved over the years to include combinations of different therapies that are more effective when administered together; however, the development of drug resistance is still common. Numerous preclinical and clinical studies are underway to identify new therapeutic combinations that disrupt the ability of CRC cells to adapt and survive by simultaneously targeting multiple pathways. Strategies specifically designed to overcome resistance mechanisms include co-administering drugs that work by different molecular mechanisms or co-treating with a drug that blocks a known mechanism of resistance. For instance, combining cytotoxic chemotherapy drugs with molecular-targeted inhibitors can have synergistic effects that circumvent resistance pathways [49]. A recent study systematically evaluated clinically relevant two-drug combinations in cancer cell lines and identified synergistic effects between chemotherapy agents and drugs targeting apoptotic signaling or cell cycle inhibitors in distinct molecular subpopulations [236]. In the context of CRC, the study found that irinotecan and CHK1 inhibition had a synergistic anticancer effect in microsatellite stable (MSS) or *KRAS-TP53* double-mutant colon cancer cells. Alternatively, combining two or more molecular-targeted therapy drugs may be used to overcome resistance due to aberrant activation of signaling pathways. For example, in *BRAF*-mutated CRC, the response to single-agent BRAF inhibition is limited by EGFR-mediated adaptive feedback reactivation of MAPK signaling [237]. However, the triple combination of BRAF, EGFR, and MEK inhibition has shown higher response rates and improved efficacy in patients with *BRAF*-mutant CRC [238,239].

Another approach that has emerged as a potential strategy to overcome resistance is the integration of immunotherapy with conventional treatments. For instance, several clinical studies are evaluating ICIs and the anti-EGFR mAb cetuximab as a therapeutic combination in CRC since cetuximab can modulate the immune composition of the TME, potentially making cancer cells more susceptible to ICIs [240]. In addition, dual immunotherapies are being investigated, including two ICI combinations in patients with MSI-H/dMMR metastatic CRC to overcome primary resistance to ICI monotherapy [241].

## 5. Conclusions and Future Perspectives

CRC is the second most lethal cancer worldwide, due in part to high rates of recurrence and eventual treatment failure. Overcoming drug resistance in CRC remains a significant challenge, requiring a multifaceted approach that involves advancements in molecular biology research and new therapeutic strategies. CRC heterogeneity and the myriad of resistance mechanisms make it unlikely that one type of therapy can eliminate all CRC cells, thus underscoring the need to develop new combination therapy approaches. Current studies emphasize the importance of targeting key signaling pathways, such as EGFR/RAS/RAF/ERK, PI3K/AKT/mTOR, and Wnt/β-catenin, which are central in CRC development and resistance mechanisms. In addition, the TME and drug-resistant subpopulations like CSCs and DTPs play crucial roles in mediating resistance, thus highlighting the need for therapies that can modulate the TME and eradicate CSCs and DTPs. The continued development of immune-based approaches, including ICIs, BiTEs, and oncolytic viruses, holds significant potential for achieving treatments that are more effective in the long term.

The integration of different emerging therapeutic approaches, such as immunotherapies, epigenetic therapies, and nanomedicine, holds the most promise for enhancing drug efficacy and helping to overcome resistance. Additionally, the repurposing of various clinically approved drugs, which were originally used to treat different diseases but also possess the ability to combat therapy resistance in CRC, can reduce the cost and time needed for drug development [148,242]. Further research into the molecular mechanisms of resistance in CRC is essential for the development of effective combination therapies that can improve treatment response and bypass or prevent resistance. It is likely that multiple mechanisms will need to be targeted to successfully overcome drug resistance, and adaptive therapy approaches could be used to adjust treatments based on tumor responses. The development of precision medicine approaches that are guided by genomic, epigenomic, and molecular profiling of CRC tumors will enable future tailoring of treatments to individual patients. Furthermore, applying biomarker-based strategies to direct therapies to patient subgroups that are most likely to benefit and monitor treatment response could substantially improve patient outcomes.

## Data Availability

Not applicable.
